# Evaluation and comparison of ocular biometric parameters obtained with Tomey OA-2000 in silicone oil-filled aphakic eyes

**DOI:** 10.1186/s12886-023-02962-w

**Published:** 2023-05-16

**Authors:** Yongqun Xiong, Yongdong Lin, Zifeng Zhao, Hongxi Wang, Guihua Zhang

**Affiliations:** Joint Shantou International Eye Center of Shantou University, The Chinese University of Hong Kong, Dong xia Road, Shantou, Guangdong Province People’s Republic of China

**Keywords:** Ocular biometry, Silicone oil-filled eyes, Retinal disease, Swept-source optical coherence tomography

## Abstract

**Purpose:**

To evaluate a new non-contact instrument (OA-2000) measuring the ocular biometry parameters of silicone oil (SO)-filled aphakic eyes, as compared with IOLMaster 700.

**Methods:**

Forty SO-filled aphakic eyes of 40 patients were enrolled in this cross-sectional clinical trial. The axial length (AL), central corneal thickness (CCT), keratometry ((flattest keratometry) Kf and (steep keratometry, 90° apart from Kf) Ks), and axis of the Kf (Ax1) were measured with OA-2000 and IOLMaster 700. The coefficient of variation (CoV) was calculated to assess the repeatability. The correlation was evaluated by the Pearson coefficient. Bland-Altman analysis and paired *t* test were used to analyze the agreements and differences of parameters measured by the two devices, respectively.

**Results:**

The mean AL obtained with the OA-2000 was 23.57 ± 0.93 mm (range: 21.50 to 25.68 mm), and that obtained with the IOLMaster 700 was 23.69 ± 0.94 mm (range: 21.85 to 25.86 mm), resulting in a mean offset of 0.124 ± 0.125 mm (*p* < 0.001). The mean offset of CCT measured by OA-2000 and IOLMaster 700 was 14.6 ± 7.5 μm (*p* < 0.001). However, the Kf, Ks and Ax1 values from the two devices were comparable (*p* > 0.05). All the measured parameters of the two devices showed strong linear correlations (all r ≥ 0.966). The Bland-Altman analysis showed a narrow 95% limits of agreement (LoA) of Kf, Ks and AL, but 95%LoA of CCT and Ax1 was wide, which were − 29.3 ~ 0.1 μm and-25.9 ~ 30.7°respectively. The CoVs of the biometric parameters obtained with OA-2000 were lower than 1%.

**Conclusion:**

In SO-filled aphakic eyes, the ocular parameters (including AL, Kf, Ks, Ax1, and CCT) measured by the OA-2000 and IOLMaster 700 had a good correlation. Two devices had an excellent agreement on ocular biometric measurements of Kf, Ks and AL. The OA-2000 provided excellent repeatability of ocular parameters in SO-filled aphakic eyes.

## Introduction

Vitrectomy with silicone oil (SO) tamponade is often needed during surgery for many serious fundus diseases, such as severe retinal detachment. For this kind of patient, accurate ocular biometry is essential for implanting an intraocular lens (IOL) with ideal refractive power, providing a chance of simultaneous operation with the SO removal [1, 2]. Immersion ultrasound biometry has been used in axial length (AL) measurement for SO-filled eyes [3]. However, this technique is difficult and operator-dependent, and the result is disturbed by the amount of SO tamponade due to the prostrate position [4, 5]. In 1999, the first optical biometer, the partial correlation interference (PCI)-based IOLMaster (Carl Zeiss Meditec AG, Jena, Germany), came into being [6]. Compared with A-scan applanation ultrasound measurement, the optical measurement is more accurate and easier to operate [7]. The PCI-based IOLMaster has become the standard for measuring ocular parameters [8–10].

With the development of technology, a new generation of biometer (OA-2000) using swept-source optical coherence tomography (SS-OCT) appears. It has a faster scanning speed and more accurate measurements [11]. Previous articles showed that the repeatability and reproducibility of OA-2000 were excellent for biometrical parameters, and high agreement between OA-2000 and PCI-based IOLMaster for most biometrical parameters in healthy subjects [11, 12]. However, the accuracy of ocular parameters obtained with the OA-2000 for SO-filled aphakic eyes has not been researched.

Therefore, the purpose of this study was to assess the repeatability of ocular parameters of SO-filled aphakic eyes measured by the OA-2000 and to compare these measurements with those provided by the IOLMaster 700.

## Materials and methods

A total of 40 SO-filled aphakic eyes of 40 patients were included in this cross-sectional study, conducted between November 2021 and June 2022. The exclusion criteria included (1) patients with previous refractive surgery, glaucoma surgery, previous episcleral surgery or patients who underwent SO tamponade twice; (2) patients with retinopathy that may influence the AL measurement, such as macular hole, or macular pucker; (3) other ocular diseases, such as nystagmus, keratopathy, ocular surface diseases, glaucoma, uveitis, ocular trauma, or strabismus. When both eyes of a subject were eligible, one eye was selected at random to avoid potential interocular correlation. The measurements were performed by the same experienced examiner. Three consecutive AL, central corneal thickness (CCT) and corneal curvature were obtained with IOLMaster 700 (Carl Zeiss Meditec, Jena, Germany) (software V.1.88) using the built-in setting. The OA-2000 (Tomey Corporation, Japan) (software V.4E) automatically performed 10 consecutive measurements for each parameter, and obtained the mean value. The anterior chamber depth (ACD) cannot be measured in aphakic patients. When the patient was examined, both instruments were set by selecting the SO-filled aphakic eyes program. Every patient was examined by OA-2000 and IOLMaster 700 on the same day, in computer-generated random order. Besides, all eyes received systematic ophthalmic examination, including subjective refraction, non-contact tonometry, slit lamp examination, and ophthalmoscopy without mydriatics. The distance of subjective refraction was 6 m, which was the standard distance suggested by Hoffer and Savini in their previous paper [13, 14].

The study was approved by the Ethics Committee of Joint Shantou International Eye Center (JSIEC) of Shantou University and the Chinese University of Hong Kong (Shantou city, China), and it followed the tenets of the Declaration of Helsinki. All participants were informed about the nature and the procedure of the research, and provided written informed consent for measurement and data analysis. Ethical approval number: EC20190612(3) -P13. Because the AL was the most crucial data, the sample size was calculated based on the AL. The sample size of the agreement was calculated according to the following formula, where n is the sample size, s is the standard deviation (SD) of the differences in AL measurement, and LoA is the limits of agreement.

$$\displaylines{1.96\sqrt {\frac{{3{s^2}}}{n}} = the{\text{ }}desired{\text{ }}confidence \cr\,\,\,\,\,\,\,\,\,\,\,\,\,\,\,\,\,\,\,\,\,\,\,\,\,\,\operatorname {int} erval{\text{ }}of{\text{ }}the{\text{ }}Lo{A}{\text{ }} \cr}$$

[1]. 

The desired confidence interval of the LoA was 0.07 mm, S was 0.12 mm (preliminary experiment acquisition), and the sample size was found to be 34.

### Instruments

The OA-2000 utilizes Fourier domain technology, with a scanning laser wavelength of 1060 nm and a scanning speed of 1000 times/s^[16, [17]]^. The signal-to-noise ratio with this technology is better than with the broadband light source because the reflections of the narrow-bandwidth wavelength light source are projected to the eye at a time^[[18], 19]^which improves tissue penetration and image quality [18]. The SS-OCT-based OA-2000 is superior to the principle of PCI, and it can use a 3D eye tracking system to automatically capture measurements [17]. Moreover, 10 scans are performed continuously in one measurement, and the visual axis does not need to be readjusted [11]. When measuring the corneal curvature, the Placido disc-based topography technique is adopted, which can be projected onto the cornea in a 5.5 mm area, with 9 rings, each ring having 256 points, and the keratometry (K) with a diameter of 2. 5 mm is obtained. Once the measurement starts, the instrument automatically scans the AL, CCT, K ((flattest K) Kf and (steep radius, 90° apart from Kf) Ks), and axis of the Kf (Ax1). When a flicker is detected during measurement, it will delete inappropriate data and automatically rescan. All the subjects were asked to sit in front of the instrument, put their chin on the chin rest, focus on one target and keep their eyes open without blinking according to the instruction manual.

The principle of the IOLMaster 700 has been described elsewhere before [19–22]. This instrument also utilizes SS-OCT technology, and the length parameters on the visual axis are obtained using a scanning light source with an average wavelength of 1055 nm, including CCT, AL [19].

### Statistical analysis

We used SPSS (ver. 22.0; SPSS Inc, Chicago, IL) for statistical analysis. The repeated measurements were used to calculate the coefficient of variation (CoV) to judge the repeatability of measurements for each device. We used the Shapiro-Wilk test to evaluate the normal distribution of continuous variables. Pearson correlation analysis was used to analyze the correlation between the AL, CCT, and corneal curvature of the two devices. Paired *t* test was used to evaluate the differences between the results of the two devices. Bland Altman analysis was used to evaluate the consistency of parameters which was performed using MedCalc software (ver.15.2.2, Mariakerke, Belgium), and 95% LoA was determined as the mean difference ± 1.96 SD of the difference. Narrower 95% LoA means higher agreement [23]. *P* < 0.05 was statistically significant.

## Results

Forty SO-filled aphakic eyes of 40 patients (18 males, accounting for 45%) with an average age of 54.9 ± 10.3 years (range, 29–74 years old) were included. Among the primary diseases, 33 eyes had complicated retinal detachment, 6 eyes had diabetic retinopathy with vitreous hemorrhage, and 1 eye had endophthalmitis. All eyes were filled with SO (Oxane 5700 Bausch & Lomb, Kingston-upon-Thames, UK) by the same specialist (GZ). Table 1 summarizes the mean values, SDs, measurement ranges, CoV and Person correlation coefficient of AL, K (Kf and Ks), Ax1, and CCT. Table 2 summarizes the differences of the means, with their SDs, 95% LoA, 95% confidence intervals and paired *t* test results for AL, K (Kf and Ks), Ax1, and CCT.

**Table 1:** Summary of results for the measured variables in both tested devices

	Device	Mean ± Standard Deviation	Range (Minimum–Maximum)	CoV (%)	Pearson correlation coefficient *(r*)
Axial length (mm)	OA-2000	23.57 ± 0.93	21.50-25.68	0.042	0.991
	IOLMaster	23.69 ± 0.94	21.85–25.86	0.029	
Keratometry of flat (D)	OA-2000	43.16 ± 1.63	40.42–47.47	0.093	0.980
	IOLMaster	43.09 ± 1.60	40.37–47.43	0.023	
Keratometry of steep (D)	OA-2000	44.48 ± 1.73	41.01–48.28	0.115	0.987
	IOLMaster	44.40 ± 1.73	40.80-48.33	0.137	
Axis of the flattest radius (°)	OA-2000	108.8 ± 51.6	4-179	0.934	0.966
	IOLMaster	106.4 ± 55.2	1-179	1.450	
Central corneal thickness (µm)	OA-2000	530.7 ± 30.9	470–578	0.412	0.978
	IOLMaster	545.3 ± 33.9	479–600	0.811	

CoV, coefficient of variation

**Table 2:** The differences of the means, with their SDs, 95% confidence intervals and paired t test results between the OA-2000 and the IOLMaster700

	Mean difference ± SD	95%LoA	95% Confidence Intervals of Differences	P
Axial length (mm)	-0.124 ± 0.125	-0.37 to 0.12	-0.164 to -0.084	< 0.001
Keratometry of flat (D)	0.075 ± 0.323	-0.56 to 0.71	-0.029 to 0.178	0.152
Keratometry of steep (D)	0.087 ± 0.275	-0.45 to 0.63	-0.001 to 0.175	0.052
Axis of the flattest radius (°)	0.15 ± 34.36	-25.9 to 30.7	-2.2 to 7.0	0.300
Central corneal thickness (µm)	-14.6 ± 7.5	-29.3 to 0.1	-17.0 to -12.2	< 0.001

Difference of the means is the value measured with the OA-2000 minus value measured with the IOLMaster 700

LoA, limits of agreement

The mean AL obtained with the OA-2000 was 23.57 ± 0.93 mm (range: 21.50 to 25.68 mm), and that obtained with the IOLMaster 700 was 23.69 ± 0.94 mm (range: 21.85 to 25.86 mm), resulting in a mean offset of 0.124 ± 0.125 mm (*p* < 0.001). The mean offset of CCT measured by OA-2000 and IOLMaster 700 was 14.6 ± 7.5 μm (*p* < 0.001). However, the Kf, Ks and Ax1 values from the two devices were comparable (*p* > 0.05). All the measured parameters of the two devices showed strong linear correlations (all r ≥ 0.966). The CoVs of all biometric parameters obtained with OA-2000 were lower than 1%. The CoVs of the biometric parameters obtained with IOLMaster 700 were lower than 1% except for the Ax1.

**Fig. 1:**
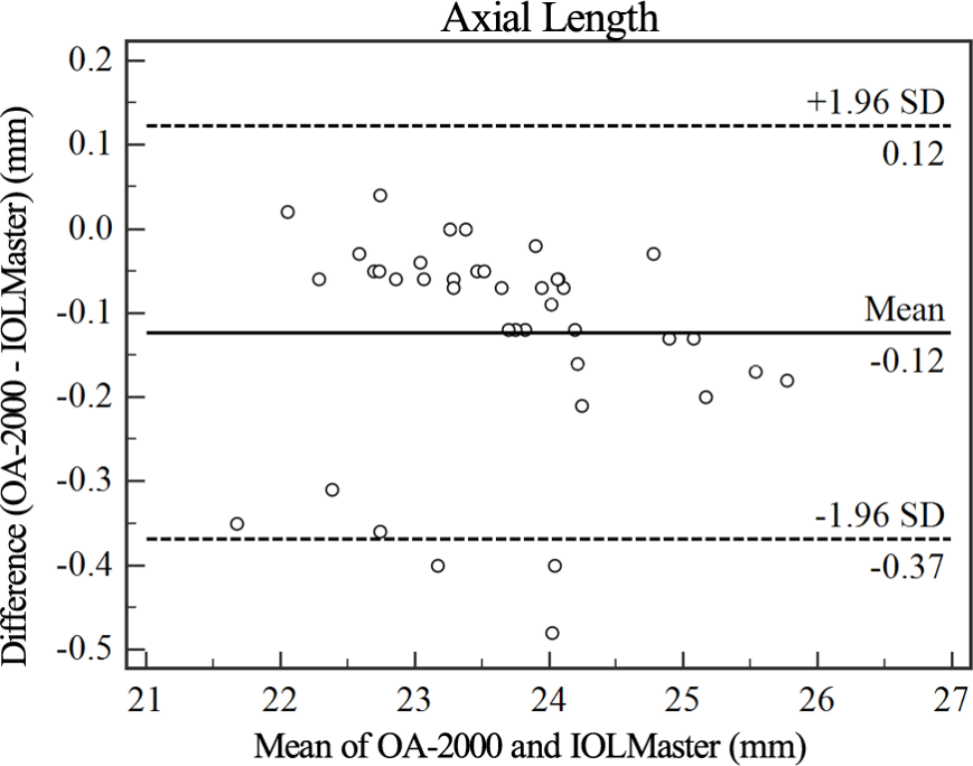
Bland-Altman plot of the axial length measurements using the OA-2000 against IOLMaster 700. The solid line represents the mean difference. The dotted lines on the side represent the upper and lower 95% limits of agreement

**Fig. 2:**
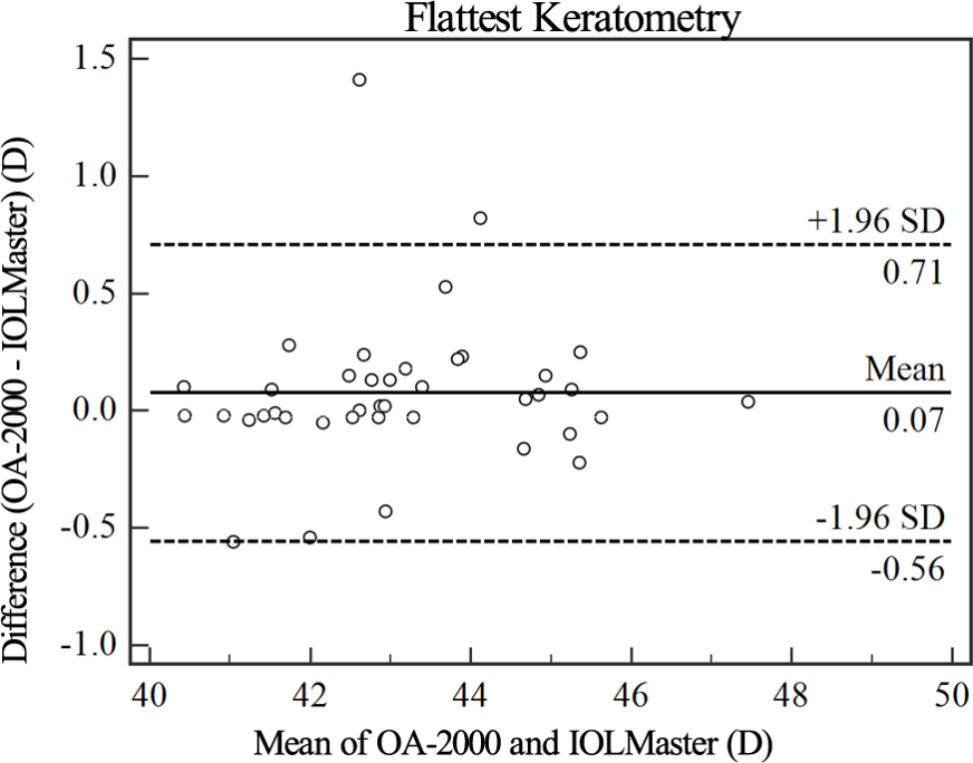
Bland-Altman plot of the flattest keratometry measurements using the OA-2000 against IOLMaster 700. The solid line represents the mean difference. The dotted lines on the side represent the upper and lower 95% limits of agreement

**Fig. 3:**
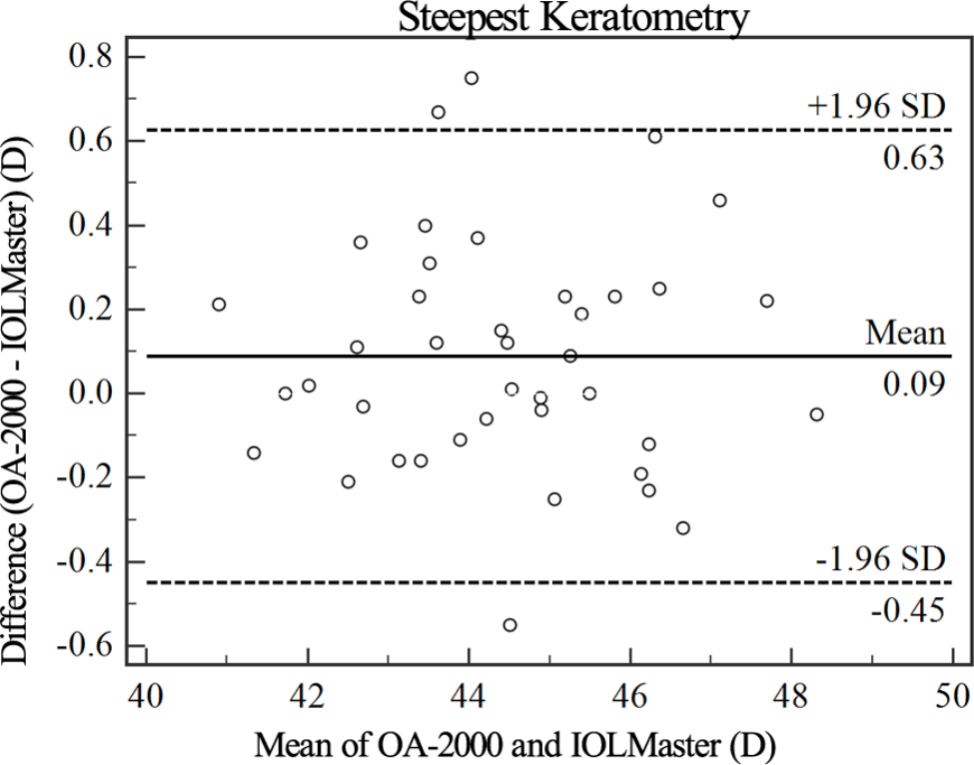
Bland-Altman plot of the steepest keratometry measurements using the OA-2000 against IOLMaster 700. The solid line represents the mean difference. The dotted lines on the side represent the upper and lower 95% limits of agreement

**Fig. 4:**
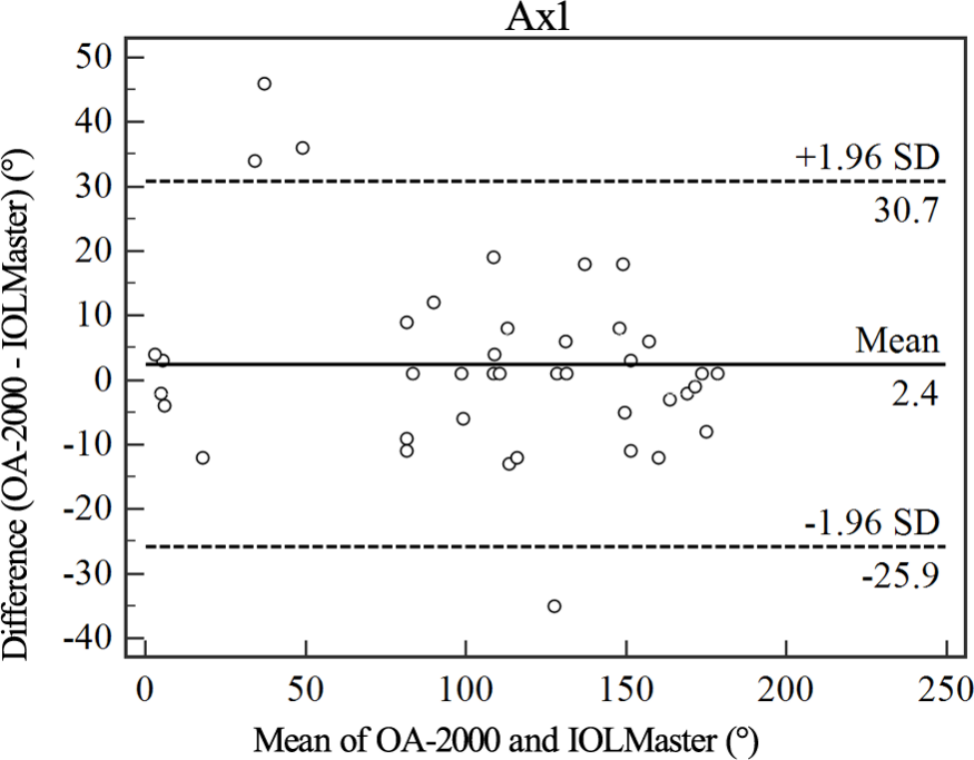
Bland-Altman plot of the axis of the flattest keratometry measurements (Ax1) using the OA-2000 against IOLMaster 700. The solid line represents the mean difference. The dotted lines on the side represent the upper and lower 95% limits of agreement

**Fig. 5:**
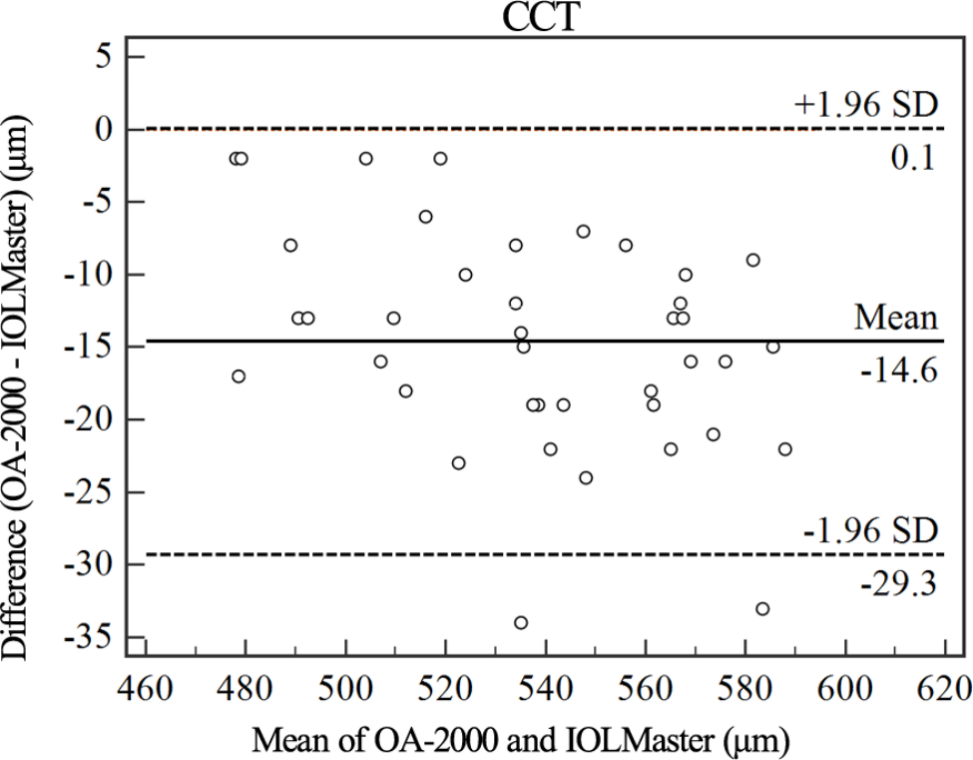
Bland-Altman plot of the central corneal thickness (CCT) measurements using the OA-2000 against IOLMaster 700. The solid line represents the mean difference. The dotted lines on the side represent the upper and lower 95% limits of agreement

Figures 1, 2, 3, 4 and 5 show Bland-Altman diagrams of AL, Kf, Ks, Ax1 and CCT measured by OA-2000 and IOLMaster 700. Bland-Altman analysis showed a narrow 95%LoA of Kf, Ks and AL, but 95%LoA of CCT and Ax1 was wide, which were − 29.3 ~ 0.1 μm and-25.9 ~ 30.7°respectively.

## Discussion

Vitrectomy and SO tamponade are usually performed on eyes that have suffered from severe eye diseases, as shown in the current study. Accurate biometrical measurement before IOL implantation is crucial for such eyes to ensure good visual outcomes. Inaccurate measurement of AL and K can lead to 36% and 22% errors in predicting IOL refraction using optical biometrics, respectively [24].

Previous studies proved that OA-2000 and IOLMaster showed high intra-session consistency in most parameters (including AL [12, 25, 26], K [12, 26], ACD [26]) of normal subjects. Huang et al. [16] used OA2000 to measure the ocular parameters of normal subjects and compared it with the PCI-based IOLMaster, which proved that OA-2000 had good repeatability and reproducibility for the parameters including AL, ACD, K, lens thickness, and corneal diameter values. Reitblat et. [27] showed that the advantage of OA-2000 was the high success rate for AL measurement. Among the 6465 patients, the AL of 301 eyes that could not be measured by IOLMaster 500 was successfully measured by OA-2000 in 284 eyes (94.35%), and the success rate for AL measurement by OA-2000 was 99.7%. However, measuring AL in SO-filled eyes brings additional challenges, such as artifacts caused by oil droplets, which can complicate the measurements. Furthermore, insufficient SO filling can result in deviations in the results, particularly in immersion ultrasound biometry where patients are in a prostrate position [4, 28]. The CoV can be used to indicate the accuracy and repeatability of measurement [5]. A lower CoV value indicates higher precision. Auffarth et al. ^5^used Pentacam AXL and IOLMaster 700 to measure the AL of pseudophakic SO-filled eyes and showed that the CoVs of Pentacam AXL and IOLMaster 700 were 0.26% and 0.09% respectively. The CoV of IOLMaster 700 was similar to our results (0.029%). Our research showed that the CoVs of the ocular parameters (including AL, Kf, Ks, Ax1, and CCT) measured by OA-2000 were lower than 1%, indicating that OA-2000 could still provide high repeatability despite more difficult conditions.

For SO-filled eyes, previous studies showed that the PCI-based IOLMaster was accurate in measuring the AL. Parravano et al. [29] measured the AL of 10 SO-filled eyes before and one week after SO removal. They found no statistical difference in the AL of SO-filled eyes before and after surgery, and IOLMaster demonstrated good accuracy in measuring the AL. Kunavisarut et al. [30] measured 34 cataractous SO-filled eyes with IOLMaster and immersion A-ultrasound before and 3 months after SO removal combined with cataract surgery and IOL implantation. They found that the AL measurement by IOLMaster also was more accurate than A-ultrasound by Pearson’s correlation (0.966 vs. 0.410). The error of A-ultrasound in predicting postoperative refraction (average 1.79 ± 1.04d) was higher than that of IOLMaster (average 0.60 ± 0.23d), with a statistically significant difference (P = 0.049). Similarly, El-Baha et al. [28] used IOLMaster and A-ultrasound to measure the AL of 22 cataractous SO-filled eyes, which underwent removing SO, cataract removal and IOL implantation. Unlike Kunavisarut et al.’s research, in El-Baha et al.’s research, the AL was measured after removing SO during operation. There was no statistical difference between the two instruments in measuring the AL, and the two instruments showed a small predictive postoperative refractive error. However, El-Baha et al.’s article did not explain whether A-scan ultrasonic biometer used contact or immersion technique. The contact technique is very unreliable and should not be used, and the immersion technique is the only ultrasound technique that should be used. The immersion technique could be useful in the case of dense cataracts [31, 32]. As mentioned above, at present, the measurement of cataract patients by any optical biometer cannot be 100% detected.

The current research showed that the AL of OA-2000 and IOLMaster 700 had good correlation and consistency in SO-filled eyes. Our study revealed a slight difference in measuring the AL between the device, with the OA-2000 measuring an average of 0.124 mm shorter than that measured by IOLMaster 700. However, the difference was clinically acceptable since, according to the SRK-T formula, an error of 0.12 mm in AL results in an error of 0. 30 ~ 0.51D [33]. On the one hand, this difference may be caused by the different algorithms or analysis programs of the boundary measured by the two devices. On the other hand, it comes from different group refractive indices (GRI) [34, 35]. Compared with vitreous, the refractive index of SO is higher, leading to a decrease in the speed of light passing through SO [36]. Previous studies showed that IOLMaster 700 and OA-2000 had little difference and good consistency in measuring the AL of normal subjects, which was consistent with our research in SO-filled aphakic eyes [25, 37]. Huang et al. ^25^compared the AL measurement and success rate of cataract patients using two types of optical biometers based on SS-OCT (IOLMaster 700, OA-2000 and Argos) and PCI (IOLMaster v 5.4). Their findings revealed that three SS-OCT biometers had significantly higher success rates in measuring the AL of the eyes with various degrees of lens opacity compared with IOLMaster v5.4. Additionally, the three SS-OCT biometers had high consistency with IOLMaster v5.4 in AL measurement. Compared with IOLMaster 700, OA-2000 measured an average of 0.01 mm shorter in AL, which was not clinically significant. Goebels et al. ^37^used OA- 2000, IOL Master 500 and Lenstar 900 devices to measure the AL in cataract patients, and found good correlation between the three instruments. The differences between OA-2000, IOLMaster 500 and Lenstar 900 in measuring the AL were quite small (0. 05 mm and 0. 03 mm respectively), which was statistically significant but not clinically significant. In addition, their research also showed that OA-2000 and IOL Master 500 were in good agreement in measuring AL and K values, which was consistent with our research in SO-filled aphakic eyes.

Our study also showed that K values of OA-2000 and IOLMaster 700 had good correlation and consistency in SO-filled eyes, while CCT had poor consistency. These results were consistent with the findings of previous studies [11, 12]. Hua et al. [11] used OA-2000 and PCI-based IOLMaster to measure 108 healthy subjects. They reported that the CoV values of K and CCT measured by OA-2000 were less than 1%. The K had good correlation and consistency between the two devices, but CCT had poor consistency. Similarly, in eyes with high myopia, Du et al. [38] studied 67 cataract eyes with high myopia, and found that the K value measured by OA-2000 and IOLMaster 500 had good correlation and consistency. Liao et al. [12] used OA-2000 and IOLMaster700 to measure the ocular parameters of 103 healthy eyes, and found a significant difference in CCT value between the two devices. The average value measured by OA-2000 was 17.08 μm less than that by IOLMaster 700, and the consistency of CCT measured by the two instruments was poor, which was consistent with our research. The average difference of CCT measured in our study was 14.6 μm. More attention should be paid to this difference in clinical application.

The limitation of this study was that we didn’t use instruments to predict the refractive error of SO-filled eyes after SO was removed and IOL implantation. Next, we will conduct research. Second, our study did not evaluate OA-2000 in measuring SO-filled eyes with cataracts. Cataract extraction with SO tamponade is necessary for patients with severe cataracts, since previous studies have shown that about 10% and 20% of eyes with severe cataracts couldn’t be measured by PCI-based optical biometer [10, 39]. Therefore, the patients enrolled in this study were aphakic SO-filled eyes. However, as reported, the success rate of OA-2000 for measuring AL had greatly improved in various degrees of lens opacities [27]. This is important for SO-filled eyes with cataracts, which can help reduce some postoperative complications after combined surgeries of anterior and posterior ocular segments. The success rate and accuracy of OA-2000 for cataract SO-filled eye measurement need further study in the future. Finally, the sample size of our study was relatively small. Our sample size was calculated based on the AL, which might not be enough for other parameters. Furthermore, McAlinden et al. [15] in their previous articles recommended that the sample size of articles (comparison of clinical tests) related to agreement and precision should be above 100.

In a word, the first clinical comparative study between OA-2000 and IOLMaster 700 in SO-filled aphakic eyes showed that Al, Kf, and Ks had good correlation and consistency between the two devices. The CoVs of the ocular parameters (including AL, Kf, Ks, Ax1, and CCT) measured by OA-2000 were lower than 1%. This study proves the possibility that OA-2000 can reliably and repeatedly obtain the most important parameters needed for IOL implantation in patients with SO-filled aphakic eyes.

## Data Availability

The author confirms that all relevant data are included in the article. The original data that support the findings of this study are available from the corresponding author on reasonable request.
